# Microvascular Density, Endothelial Area, and Ki-67 Proliferative Index Correlate Each Other in Cat Post-Injection Fibrosarcoma

**DOI:** 10.3390/cells10010031

**Published:** 2020-12-28

**Authors:** Rosa Patruno, Giuseppe Passantino, Carmelo Laface, Antonella Tinelli, Alfredo Zito, Roberta Ruggieri, Francesco Luposella, Pietro Gadaleta, Mariarita Laforgia, Luca Lacitignola, Michele Ammendola, Girolamo Ranieri, Nicola Zizzo

**Affiliations:** 1 Department of Veterinary Medicine, Section of Veterinary Pathology and Comparative Oncology, University of Bari “Aldo Moro”, Strada p.le per Casamassima, km 3, 70010 Valenzano, Bari, Italy; rosavet@libero.it (R.P.); giuseppe.passantino@uniba.it (G.P.); antonella.tinelli@uniba.it (A.T.); nicola.zizzo@uniba.it (N.Z.); 2 Interventional and Medical Oncology Unit, IRCCS Istituto Tumori “G. Paolo II”, 70124 Bari, Italy; carmelo.laface@gmail.com (C.L.); cd.gadaleta@gmail.com (P.G.); 3 Department of Biomedical Sciences and Clinical Oncology, University of Bari Aldo Moro, 10124 Bari, Italy; 4 Pathology Unit, IRCCS Istituto Tumori “G. Paolo II”, 70124 Bari, Italy; fazito@libero.it; 5 Cardiology Unit, University of Bari “Aldo Moro”, 70124 Bari, Italy; roberta.ruggieri@gmail.com; 6 Direction Départementale de la Cohésion Sociale et de la Protection des Populations des VOSGES (DDCSPP88), 88080 Vittel, France; francescoluposella@hotmail.it; 7 Pharmacy Unit, IRCCS Istituto Tumori “G. Paolo II”, 70124 Bari, Italy; m.laforgia@oncologico.bari.it; 8 Department of Emergency and Organ Transplantation, University of Bari ‘Aldo Moro’, Strada p.le per Casamassima, km 3, 70010 Valenzano, Bari, Italy; luca.lacitignola@uniba.it; 9 Department of Health Science, Digestive Surgery Unit, University “Magna Graecia” Medical School, Viale Europa, Germaneto, 88100 Catanzaro, Italy; michele.ammendola@libero.it

**Keywords:** angiogenesis, cat fibrosarcoma, endothelial area, immunohistochemistry, Ki-67 proliferation index, microvascular density

## Abstract

Soft tissue sarcomas are a large group of different tumor types both in humans and in animals. Among them, fibrosarcoma is the most frequent malignant mesenchymal tumoral form in cats, representing up to 28% of all cat skin tumors, while human fibrosarcoma, fortunately, only represents 5% of all sarcomas and 0.025% of the world-wide burden of tumors. This low incidence in humans leads to consideration of this group of tumoral diseases as rare, so therapeutic options are few due to the difficulty of starting clinical trials. In this context, the identification of research models for fibrosarcomas could be of great interest to deepen knowledge in this field and recognize new or possible biological pathways involved in tumor progression and metastasis. Angiogenesis is considered a fundamental scattering cause of tumor aggressiveness and progression in all forms of cancer, but only a few research parameters were developed and reported to express them quantitatively and qualitatively. The role in angiogenesis of microenvironmental stromal cells, such as fibroblasts, lymphocytes, mast cells, and macrophages, was largely demonstrated since this topic was first approached, while quantification of new vessels and their blood capacity in tumoral area is a relatively recent approach that could be well developed thanks to expertise in immunohistochemistry and image analysis. In this paper, a crossing study evaluating microvascular density (MVD), endothelial area (EA), and Ki-67 proliferative index was reported for a series of formalin-fixed and paraffin-embedded tissue samples from 99 cat patients, affected by cat post-injection fibrosarcoma, by using a till ×400 magnification light microscopy. We aim to demonstrate that cat pets may be considered a useful animal model for better studying the correspondent human diseases and we report, for the first time to our knowledge, experimental data in terms of correlation among MVD, EA, and Ki-67 strictly involved in aggressiveness and tumoral progression.

## 1. Introduction

Pet animals, cats and dogs, have been largely used as models for oncological research since the spread of the “one medicine and pathology approach” to cancer [1,2]. With special regard to soft tissue tumors, cat post-injection fibrosarcoma (CPIFI) is certainly a useful model to deepen knowledge about sarcomas [3,4,5,6]. From a clinical point of view, fibrosarcoma (FI), which originates from fibroblasts, is the most common malignant mesenchymal tumor in cats, representing 14% to 28% of all cat skin tumors, with an onset average age of 12 years [7]. Subcutaneous FIs frequently arise on the trunk and distal parts of the limbs, while dermal FIs hit the pinnae and digits in most cases [8], but these tumors often recur and metastasize to distant organs, inducing death of the animal [9]. To perform their metastatic ability, sarcoma cells need to penetrate blood vessels and, in turn, proliferate in the chosen metastatic site. An increased microvascular bed, depicted by the angiogenesis processes, facilitates the metastatic spread [10]. With special regard to CPIFI, its sprouting at the vaccination site was previously reported [8,11]. From a pathogenetic point of view, literature data correlate stromal infiltrate cells to CFI development [12,13], underlining that microenvironmental stromal cells, such as fibroblasts, lymphocytes, mast cells, and macrophages, sustain angiogenesis in several human and animal malignancies [14,15,16,17,18,19,20,21,22,23,24]. Due to its origin from fibroblasts, CPIFI has a rich stromal cell infiltrate which is supposed to be responsible for the pathogenesis of the tumor [25]. It is also noteworthy that the number of tumor cell mitoses per field was considered a parameter of the biological aggressiveness of sarcoma, as for other solid tumors [26,27]. A higher Ki-67 proliferative index, being the expression of CPIFI malignancy and aggressiveness, should be accompanied by a higher neovascularization process [16,28]. For the first time in the literature, Couto et al. evaluated the correlation between the proliferative activity, evaluated as Ki-67 positive tumoral fibroblastic cells, and angiogenesis in terms of microvascular density (MVD) in 60 CPIFIs in 2002. However, no data are published regarding the correlation between the proliferative activity and angiogenesis both in terms of microvascular density (MVD) and endothelial area (EA) as pathologic cellular pathways in CPIFI development [18,29,30]. MVD represents the number of immunostained old and new vessels, while EA is the immunostained vascular area in a microscopic field that is the expression of vessels’ blood capacity and diameters. In combination, the two research parameters fuse different quantitative aspects of angiogenesis. The main endpoint of this research was to explore this; to this aim, we studied a series of 99 CPIFIs in terms of proliferative index of tumoral fibroblastic cells and angiogenesis using immunohistochemistry and image analysis systems. Interestingly, thanks to its higher incidence compared to human FI, although its development is scattered by an inflammatory reaction to the injection site, CPIFI might be considered a spontaneous model to evaluate angiogenesis and antiangiogenesis pharmacological strategy, which could be translated to humans.

## 2. Patients and Methods

### 2.1. Patients

A series of formalin-fixed and paraffin-embedded tissue samples obtained from 99 cases of CPIFI were employed. Patients were observed at the Department of Veterinary Medicine of Bari University and, after complete clinical examination, biopsy for each cat was performed. In the next step, the cats with confirmed histological diagnosis of FI were surgically treated [31]. Table 1 shows the patients’ characteristics.

### 2.2. Hematoxylin–Eosin and Immunohistochemistry

**Figure 1:**
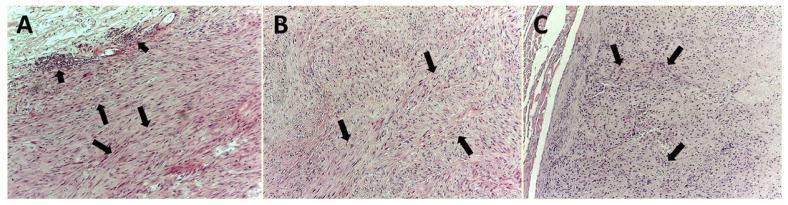
Hematoxylin and eosin staining. Magnification ×100. (**A**) Well-differentiated cat post-injection fibrosarcoma (CPIFI) section. Big arrows indicate groups of tumor cells that closely resembled the mature differentiated fibroblasts, with a low mitotic rate. (**B**) Big arrows indicate groups of tumor cells with a defined histologic phenotype and an intermediate mitotic rate. (**C**) Big arrows indicate groups of poorly differentiated tumor cells with a high mitotic rate.

Histological diagnosis was performed on serial slides for each tumor sample, stained with hematoxylin–eosin method (Figure 1A–C). According to Couto’s classification, the cases were defined as follows: 32 (32.3%) were G1, corresponding to well differentiated, 38 (38.4%) were G2, corresponding to intermediately differentiated, and 29 (29.3%) were G3, corresponding to poorly differentiated CPIFIs [26,27]. For the evaluation of MVD, EA, and Ki-67 proliferative index, a three-layer biotin–avidin–peroxidase system, as previously described, was adopted [16,18,28]. Briefly, 5 µm thin serial sections of formalin-fixed and paraffin-embedded were cut for each tissues sample. For antigen retrieval, the obtained slides were processed with a microwave oven at 500 watts for 10 min; then, the endogenous peroxidase enzyme was inhibited with a 3% hydrogen peroxide solution. Soon after, the slides were incubated with the following primary antibodies:

(a) The rabbit polyclonal anti-factor VIII-related antigen (FVIII-RA) antibody (dilution, 1:50; Dako, Glostrup, Denmark) for 1h at room temperature;

(b) The MIB-1 antibody to Ki-67 nuclear proliferation antigen (dilution, 1:100; MIB-1; Immunotech, Inc., Marseilles, France) for 1 h at room temperature.

Immunoreactivity was evidenced by employing a biotinylated secondary antibody, the avidin–biotin–peroxidase complex (LPS, K0640, Dako, Glostrup, Denmark), and the chromogen 3-amino-9-ethyl carbazole (Dako, Glostrup, Denmark). Nuclear counterstaining was performed with Gill’s haematoxylin (Polysciences, Warrington, PA, USA) for each tissue sample [32]. An additional slide from each cat without the primary anti-factor VIII antibody was used as negative control. For the Ki-67 proliferative index, sections from normal feline intestines were used for each experiment and the corresponding epithelium was considered negative if it lacked immunostaining after utilization of the MIB-1 primary antibody.

Feline granulation tissue specimens were used as a positive control for blood vessels. Regarding the MIB-1 antibody, positive controls consisted of normal feline small intestines, because intestinal crypts are considered sites of proliferation. 

### 2.3. Image Analysis

**Figure 2:**
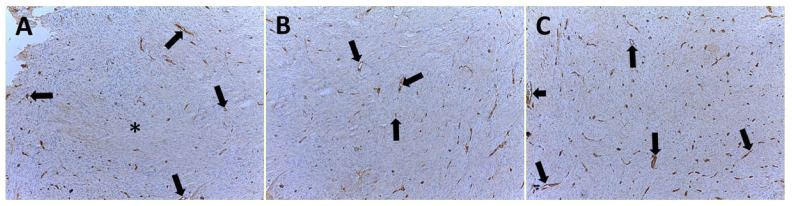
Immunohistochemistry with the primary antibody to factor VIII-related antigen, vascular “hot spots”. Magnification ×100. (**A**) Well-differentiated CPIFI section with corresponding low neovascularization. Arrows indicate brown immunostained microvessels. Note the vessel lumen in some of them. The asterisk indicates no vascularized area. (**B**) Intermediate CPIFI section with the corresponding intermediate neovascularization. Arrows indicate brown immunostained microvessels. Note the vessel lumen in some of them. (**C**) Poorly differentiated CPIFI section with the corresponding high vascularization. Arrows indicate brown immunostained microvessels. Note the vessel lumen in some of them. The small arrow indicates a microvessel with circulating cells in its lumen as an internal positive control.

MVD was identified according to the modified Weidner’s method [33]. Weidner’s method provides that any brown-staining endothelial cell or endothelial-cell cluster clearly separated from adjacent microvessels, tumor cells, and other connective-tissue elements are considered single, countable microvessels. Vessel lumens, although usually present, are not necessary for a structure to be defined as a microvessel, and red cells are not used to define a vessel lumen. Each count is expressed as the highest number of microvessels identified within any 200× or 400× field. All counts are performed by two investigators using a double-headed light microscope simultaneously; both operators agreed on what constituted a single microvessel before any vessel was included in the count [34]. In our study, we employed a modified technique; the slides were morphometrically evaluated independently by two investigators with the help of an image analysis system (Quantimet 500 Leica). To be specific, first of all, we selected vascular “hot spots” for each tumor grade at low magnification ×100 at light microscopy (Leica DM 4000). Then, ten fields were randomly selected at ×400 magnification in a 0.19 mm^2^ area (Figure 2A–C).

**Figure 3:**
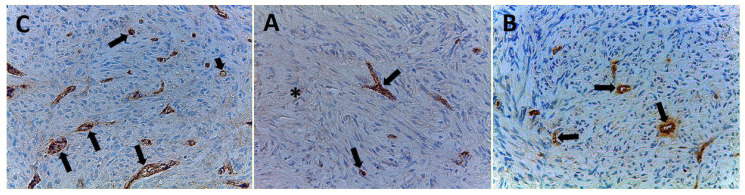
Immunohistochemistry with the primary antibody to factor VIII-related antigen. Magnification ×400. (**A**) Well-differentiated CPIFI section with the low microvascular density (MVD). Arrows indicate brown immunostained microvessels. The asterisk indicates no vascularized area. (**B**) Intermediate CPIFI section with intermediate MVD. Arrows indicate brown immunostained microvessels. Note the vessel lumen in some of them. (**C**) Poorly differentiated CPIFI section with high MVD. Arrows indicate brown immunostained microvessels. Note the vessel lumen in some of them. The small arrow indicates a microvessel with circulating cells in its lumen as an internal positive control.

Angiogenesis was also evaluated in terms of EA in a semiautomated approach, delimiting the perimeter of the single vessel and then calculating the corresponding area by the software of the image analysis system, both for each vessel and then for all vessels in the observed ×400 magnification in a 0.19 mm^2^ area (Figure 3A–C) [16,28,32,33,34,35,36,37,38]. 

**Figure 4:**
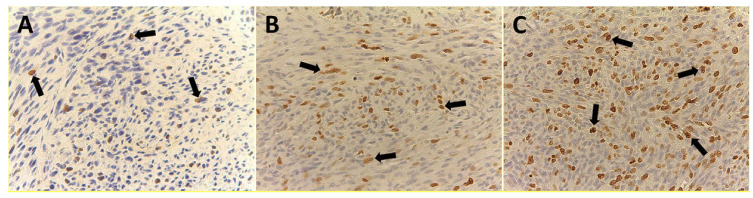
Immunohistochemistry with the primary MIB-1 antibody to Ki-67 nuclear proliferation antigen. Magnification ×400. (**A**) Well-differentiated CPIFI section with the low Ki-67 proliferation index. Arrows indicate brown immunostained nuclei. (**B**) Intermediate CPIFI section with the intermediate Ki-67 proliferation index. Arrows indicate brown immunostained nuclei. (**C**) Poorly differentiated CPIFI section with the high Ki-67 proliferation index. Arrows indicate brown immunostained nuclei.

In the serial section, Ki-67 proliferative index evaluation was determined in the same area of MVD, and only specific nuclear staining was considered. The number of MIB-1 positively stained nuclei tumor cells was counted at ×400 magnification in a 0.19 mm^2^ area (Figure 4A–C). The fraction of Ki-67 positive cells was calculated as the ratio of positively stained tumor cells and all tumor cells observed in the analyzed microscopic field [18,29,30].

All the above tissue parameters were independently evaluated by two operators (authors NZ and AZ, veterinary and human pathologists, respectively), with a correlation coefficient of ≥90%.

### 2.4. Statistical Analysis

Mean value ± standard deviations (S.D.) was evaluated for MVD, EA, Ki-67, expression in G1, G2 and G3 CPIFI subgroups. The significance of differences in terms of MVD, EA, and Ki-67 means between G1 versus (vs.) G2, G2 vs. G3, and G3 vs. G1 tumor groups was performed by one-way ANOVA test. Correlations among MVD, EA, and Ki-67 were calculated using Pearson’s (r) analysis. All statistical analyses were performed with the SPSS statistical software package (SPSS, Inc., Chicago, IL, USA).

## 3. Results

**Figure 5:**
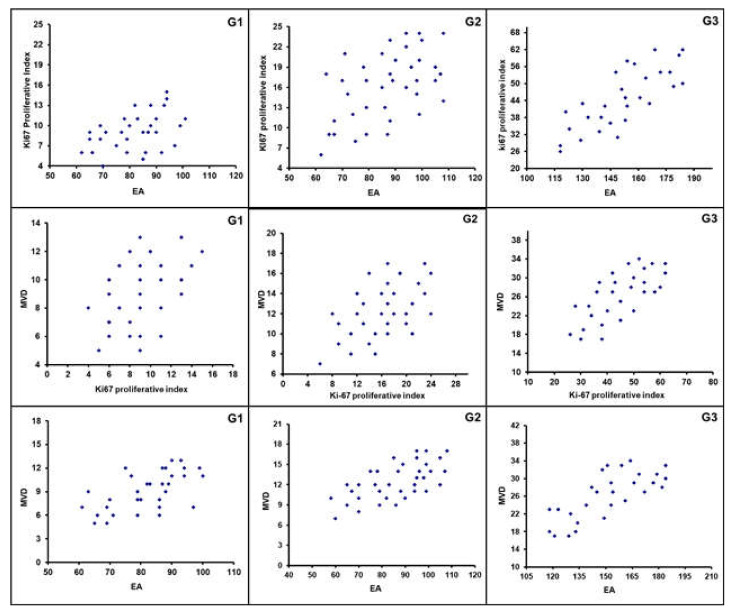
Correlation analyses between: Ki67 and MVD, G1 (r = 0.42); Ki67 and MVD, G2 (r = 0.51); Ki67 and MVD, G3 (r = 0.78); MVD and Ki67, G1 (r = 0.48); MVD and Ki67, G2 (r = 0.54); MVD and Ki67, G3 (r = 0.71); MVD and EA, G1 (r = 0.60); MVD and EA, G2 (r = 0.65); MVD and EA, G3 (r = 0.75).

All 99 samples from CPIFI were eligible for the evaluation of both MVD and EA. The FVIII-RA immunostaining showed good intensity on endothelial cells with no or low perivascular background (Figure 2 and Figure 3). Table 2 shows the mean values of MVD and EA on tissue sections of CPIFI grouped according to the Couto’s classification [26]. Significant differences in terms of MVD re demonstrated between G1 vs. G3 (9 ± 4 S.D. vs. 25 ± 9 S.D.; f = 282.80, *p* = 3.53 × 10^−24^) and G2 vs. G3 (12 ± 5 S.D. vs. 25 ± 9 S.D.; f = 196.54, *p* = 2.53 × 10^−21^) (Table 2). In the same manner, significant differences in terms of EA were evident between G1 vs. G3 (81.96 × 10^−2^ ± 20.30 μm^2^ S.D. vs. 151.77 × 10^−2^ ± 33.52 μm^2^ S.D.; f = 288.75, *p* = 2.12 × 10^−24^) and G2 vs. G3 (83.01 × 10^−2^ ± 25.48 μm^2^ S.D. vs. 151.77 × 10^−2^ ± 33.52 μm^2^ S.D.; f = 247.24, *p* = 7.79× 10^−24^) (Table 2). In the case of Ki-67-positive fraction in terms of MIB-1-positive nucleus, significant differences were found between G1 vs. G3 (9.35 ± 5.82 S.D. vs. 44.33 ± 17.91 S.D.; f = 340.83, *p* = 3.41 × 10^−26^) and G2 vs. G3 (15.16 ± 8.91 S.D. vs. 44.33 ± 17.91 S.D.; f = 214.29, *p* = 2.97 × 10^−22^) (Table 2). The above-analyzed tissue biomarkers were significantly crosslinked each other in each subgroup of malignancy grade (G1, G2, and G3 respectively, r ranging from 0.41 to 0.78, *p* ranging from 0.01 to 0.03, as shown in Figure 5).

## 4. Discussion

Soft tissue sarcomas are malignancies deriving from mesenchymal tissues and, though sharing a common origin, are a large group of different tumor types. The low frequency of human sarcomas, with an incidence of about 0.5–1% of the annual burden of all human malignancies [39,40], limits research to a very little series of cases for each histological type. In particular, human FI constitutes 5% of all sarcomas, representing 0.025% of annual burden in the world [41]. Despite the progress in multimodality treatment, the prognosis for all soft tissue sarcomas is still poor [42]. Consequently, the availability of a possible spontaneous animal sarcoma model sharing similar pathological and biological features with humans could be very useful to clarify both the angiogenetic and proliferative pathways for comparative and translational rebound [18,43,44,45,46,47,48]. To this regard, the role of angiogenesis, in terms of MVD and EA, is important for primary tumor growth, invasion and metastasis [32,37,47,49,50]. Angiogenesis is sustained by several proangiogenic factors and, among them, vascular endothelial growth factor (VEGF) is the most involved and biologically characterized, correlating with malignant development and progression in several human and animal malignancies [51,52,53,54,55,56,57,58,59]. In particular, in the in vivo preclinical study performed in fibrosarcoma HT1080-conditioned medium cell line, experimental data demonstrated that the conditioned medium expressed a higher VEGF concentration if compared to a human bone marrow-derived mesenchymal stem cell culture, used as control [60]. In addition, the injection of HT1080-conditioned medium into mouse ischemic limbs significantly induced capillary density and blood perfusion when compared with the injection of fresh medium. Interestingly, the reduction of angiogenesis, tumor growth, and metastases following the administration of the anti-VEGF antibody [61,62,63,64] in a xenograft model of human fibrosarcoma HT1080 cell line was previously reported. The involvement of angiogenesis in murine FI experimental model was also suggested by Lee et al., who demonstrated the role of alpha-Tumor Necrosis Factor in stimulating angiogenesis [65]. From a therapeutic point of view, angiostatin cDNA coding from mouse angiostatin into murine T241 fibrosarcoma cells is able to inhibit angiogenesis and tumor growth in C57Bl6/J mice, confirming the role of neovascularization in this preclinical model [53]. Finally, the simultaneous overexpression of PDGF-BB and FGF2 in murine FI led to the formation of high-density immature microvessels without pericytes, strongly suggesting the role of angiogenesis in FI models [66]. In summary, these preclinical and laboratory animal studies indicate that the angiogenetic process is one of the fundamental scattering causes for FI growth and progression [15]. In parallel, the proliferative index of tumoral cells evaluated in terms of Ki-67-immunostained nuclei by MIB-1 antibody was correlated with poor prognosis in several human and animal tumors. The above-reported data are in harmony with our results. In fact, we demonstrated that an enhanced angiogenesis, evaluated through MDV and EA values, is concomitant to tumoral increased malignancy, that is, more aggressive and proliferative tumors induce more active angiogenesis. Moreover, more undifferentiated forms develop a higher tumor ability to spread metastases.

For the first time in the literature, Couto et al. (2002) evaluated the correlation between proliferative activity in terms of Ki-67-positive tumoral fibroblastic cells and angiogenesis in terms of MVD in 60 CPIFIs [26]. However, to our knowledge, no data were reported regarding the correlation between proliferative activity and angiogenesis, both in terms of MVD and EA, as pathologic cellular pathways in CPIFI development. Both studies demonstrate with objective data that, in a FI spontaneous animal model, angiogenetic processes are directly related to malignancy degrees. This concept feeds a huge research area on angiogenetic drugs to be shared between animals and humans, with angiogenesis and mitosis growing in number related to negative prognosis, as indicated by Couto’s grading in FI. Ki-67 is a useful measure of cell mitotic activity, being higher in G3 with respect to G2 and in G3 with respect to G1 in our 99 experimental CPIFIs.

However, some differences can be found between our study and Couto’s [26]. First of all, we performed an evaluation on a wider case series (90 vs. 60 CPIFIs). Secondly, we introduced another tissue parameter as a key element of the discussion, endothelial area, which corresponds to the immunostained vascular area in a microscopic field, that is, the expression of vessels’ blood capacity and diameters. Combined data of MVD and EA give different quantitative aspects of angiogenesis. Moreover, we applied the modified Weidner’s method for the quantification of MVD (see Section 2 and Section 2.3), while Couto employed another method. Tissue sections stained with anti-CD31 antibody were scanned at 200× magnification to select the areas of highest vascularization. From these areas, three nonoverlapping fields were identified and captured using a digital camera connected to a microscope and saved on a computer. These same areas were then identified in the adjacent sections stained with factor VIII antibody and imaged in the same manner. A coloured filter was developed to allow specific detection of the chromogen on CD31- and factor VIII-stained slides. Chromogen was detected on slides using a computer and image manipulation software. With this software, pixels stained with chromogen were selected, converted to black, and transferred to a white background to create a binary image. The total number of black and white pixels in each image was quantified using an image analysis program. Vascular density was calculated by dividing the total number of black pixels by the total number of pixels within the image. Total vascular density was the mean of values obtained for three images captured from each tissue examined and was expressed as a percentage (in our study we evaluated the count directly). Neovascularization, expressed as a percentage, was determined as the absolute value of the total vascular density derived from factor VIII-rag-stained sections minus the vascular density derived from CD31-stained sections.

Regarding the Ki-67 proliferative index evaluation, there are three main differences between Couto’s method and our technique:

(1) According to Couto’s method, images of six nonoverlapping, 400× magnification fields along the periphery (growth fronts or hot spots) of the tumor were captured. Instead, we performed Ki-67 proliferative index evaluation in the adjacent sections with respect to the MVD count, selecting images of ten nonoverlapping, 400× magnification fields.

(2) According to Couto’s method, the number of fields was calculated so that a minimum of 1000 cells was counted per specimen. The total number of cells counted per field varied depending on the cellular density of each tumor. Instead, we performed the evaluation without a minimum cut-off of cells.

(3) According to Couto’s method, values obtained for positive and negative cells were summed and the proliferative fraction of peripheral regions of each tumor was determined to be the number of cells immunolabeled with MIB-1 (red) divided by the total number of cells counted per tumor. For comparison, the same procedure was applied to six nonoverlapping, 400× fields within the central region of each tumor. Instead, in our study, the fraction of Ki-67-positive cells was calculated as the ratio of positively stained tumor cells and all tumor cells observed in the analyzed microscopic field.

Our results demonstrate a statistically significant association among increased angiogenesis, proliferation, and malignancy degree in FI through concrete and objective parameters, i.e., MDV, EA, and Ki-67 values; a higher proliferation index induced increased tumoral vascularization and malignancy. These data were in agreement with our and other previously published studies on human malignancies [28,67,68]. With special regard to our data, we already demonstrated that MVD, EA, and Ki-67 proliferative index were significantly correlated to each other in pancreatic ductal adenocarcinoma patients [28].

Despite people not sharing the same high propensity for inflammation-associated sarcoma development as in feline injection-site fibrosarcoma, CPIFI has a high incidence compared to humans but shares a similar marked tendency to metastasize, in particular to the lungs [69,70]. Moreover, from a well-known biological point of view, human fibrosarcoma can grow through tumoral angiogenesis. In fact, Couto [26] already demonstrated that G3 CPIFIs, with a higher metastatic capacity, were associated with a higher angiogenesis in terms of total microvascular density with respect to G1 and G2 CPIFIs. In our study, we confirmed that MVD was higher in G3 CPIFIs with respect to G1 and G2 ones. This aspect suggests that angiogenesis can be considered an interspecies mechanism for tumor growth and progression.

Moreover, it is well known that in human fibrosarcomas, Ki-67 proliferative index is correlated with a higher malignant grade and a greater tendency to metastasize. Similarly, Couto already demonstrated that Ki-67 proliferative index is higher in G3 CPIFI with respect to G1 and G2 ones, with a greater tendency to metastasize.

In summary, the literature data demonstrated that CPIFI and human fibrosarcoma share angiogenetic and proliferative pathways for their tumor growth and progression. As a consequence, we suggest that cat FI spontaneous model might be a good preclinical background for evaluating novel treatments, combining angiogenetic inhibitors and antiproliferative chemotherapeutic drugs before further translating to humans.

## Figures and Tables

**Table 1:** Patients’ characteristics.

Variable	No. of Patients
Age years	
<8	34
>8	65
Gender	
Male	44
Female	55
Tumor site	Interscapular area (40)
	Dorsal area (21)
	Costal area (15)
	Left Scapular area (12)
	Right Scapular area (7)
	Left neck (2)
	Thigh sx (2)
Histological grade	
G1	32
G2	38
G3	29
Average time from vaccine injection to diagnosis	2.1 years

**Table 2:** MVD, endothelial area (EA), and Ki-67-positive fraction indexes means ± standard deviations analyzed as a function of CPIFI malignancy grade. The significance of differences in terms of MVD, EA, and Ki-67 means between G1 vs. G2, G2 vs. G3, and G3 vs. G1 tumor groups was performed by one-way ANOVA test.

No. of Patients (%)	MVD ×400	EA ×400	Ki-67 Index in Terms of MIB-1 Positive Nuclei ×400
G1 (32/99–32.3%)	9 ± 4	81.96 × 10^−2^ ± 20.30 μm^2^	9.35 ± 5.82
G2 (38/99–38.4%)	12 ± 5	83.01 × 10^−2^ ± 25.48 μm^2^	15.16 ± 8.91
G3 (29/99–29.3%)	25 ± 9	151.77 × 10^−2^ ± 33.52 μm^2^	44.33 ± 17.91
ANOVA test	G1 vs. G2	G1 vs. G2	G1 vs. G2
	n.s.	n.s.	n.s.
	G1 vs. G3	G1 vs. G3	G1 vs. G3
	f = 282.80	f = 288.75	f = 340.83
	*p* = 3.53 × 10^−24^	*p* = 2.12× 10^−24^	*p* = 3.41 × 10^−26^
	G2 vs. G3	G2 vs. G3	G2 vs. G3
	f = 196.54	f = 247.24	f = 214.29
	*p* = 2.53 × 10^−21^	*p* = 7.79. × 10^−24^	*p* = 2.97 × 10^−22^

## Data Availability

The data presented in this study are available on request from the corresponding author.
